# Schizophrenia-associated SLC39A8 polymorphism is a loss-of-function allele altering glutamate receptor and innate immune signaling

**DOI:** 10.1038/s41398-021-01262-5

**Published:** 2021-02-19

**Authors:** Wei Chou Tseng, Veronica Reinhart, Thomas A. Lanz, Mark L. Weber, Jincheng Pang, Kevin Xuong Vinh Le, Robert D. Bell, Patricio O’Donnell, Derek L. Buhl

**Affiliations:** 1 Wave Life Sciences, Cambridge, MA 02138 USA; 2 Biogen, Cambridge, MA 02139 USA; 3 Pfizer, Inc., Cambridge, MA 02139 USA; 4 Takeda Pharmaceuticals, Cambridge, MA 02139 USA

**Keywords:** Molecular neuroscience, Physiology

## Abstract

Schizophrenia is a complex and heterogenous disease that presents with abnormalities in glutamate signaling and altered immune and inflammatory signals. Genome-wide association studies have indicated specific genes and pathways that may contribute to schizophrenia. We assessed the impact of the functional missense variant SLC39A8 (ZIP8)-A391T (ZIP8^A391T^) on zinc transport, glutamate signaling, and the neuroinflammatory response. The ZIP8^A391T^ mutation resulted in reduced zinc transport into the cell, suggesting a loss in the tight control of zinc in the synaptic cleft. Electrophysiological recordings from perturbed neurons revealed a significant reduction in NMDA- and AMPA-mediated spontaneous EPSCs (sEPSCs) and a reduction in GluN2A and GluA1/2/3 receptor surface expression. All phenotypes were rescued by re-expression of wild-type ZIP8 (ZIP8^WT^) or application of the membrane-impermeable zinc chelator ZX1. ZIP8 reduction also resulted in decreased BBB integrity, increased IL-6/IL-1β protein expression, and increased NFκB following TNFα stimulation, indicating that ZIP8 loss-of-function may exacerbate immune and inflammatory signals. Together, our findings demonstrate that the A391T missense mutation results in alterations in glutamate and immune function and provide novel therapeutic targets relevant to schizophrenia.

## Introduction

Recent genome-wide association studies (GWAS) have improved our understanding of the genetic basis of psychiatric disorders^1^. Several independent GWAS studies have revealed genetic associations between schizophrenia and functional missense variants in the SLC39A8 gene^2–5^, which encodes the cation metal transporter ZIP8^6^. Among these human single nucleotide polymorphism (SNP) variants, non-synonymous rs13107325 presents an interesting mechanism as it encodes an amino acid change from alanine to threonine in the ZIP8 transporter at position 391 (ZIP8^A391T^)^2^. ZIP8 belongs to the Solute Carrier Family and transports divalent metal ions, including zinc and manganese, from the extracellular environment or intracellular compartments into the cytosol^6,7^. Despite the strong genetic association between *ZIP8* and schizophrenia, the role of ZIP8 and its variants in schizophrenia pathophysiology have remained largely uncharacterized.

Zinc, in its free ionic form Zn^2+^, is indispensable for all systems throughout the body, especially for immune and nervous system function. A growing body of evidence has indicated that the level of zinc is highly regulated due to its dynamic role in brain physiology. Zinc is incorporated into presynaptic vesicles via a variety of zinc transporters enriched at glutamatergic nerve terminals found in cerebral cortex, amygdala, cingulate cortex, hippocampus, and olfactory bulb^8,9^. At these synapses, zinc is co-released with glutamate into the synaptic cleft, transiently bringing interneuronal zinc concentration to micromolar levels^10–12^. This extracellular zinc profoundly affects the activity of a variety of postsynaptic ionotropic receptors, and among these targets of synaptic zinc, *N*-methyl-D-aspartate receptors (NMDARs) are rather unique as they display very high sensitivity to extracellular zinc^13,14^. In particular, the primary synaptic NMDAR subunit, GluN2A, is inhibited by nanomolar levels of zinc via an allosteric binding site located in the large bilobate N-terminal domain of the GluN2A subunit^15^. The result of zinc-mediated inhibition of GluN2A–NMDARs leads to a decrease in calcium influx and thus a decrease in neuronal activation^13,16^. Glutamate hypofunction in the prefrontal cortex has been implicated in cognitive dysfunction associated with many brain disorders, including schizophrenia, which is supported by the finding that numerous drugs blocking NMDAR receptors are known to trigger acute psychotic reactions^17,18^. Thus, alterations in zinc signaling may contribute to synaptic anomalies in schizophrenia.

In addition to the role zinc plays in regulating synaptic activity, zinc homeostasis is also important for blood–brain barrier (BBB) integrity and immune response, both of which are proposed to significantly influence many neurological conditions. In schizophrenia, disruption of the BBB and exacerbated immune responses are thought to contribute to the progression and cognitive symptoms of the disease (for review, see ref. ^19^). Recent evidence from a PheWAS study indicates that the ZIP8^A391T^ missense variant is associated with intracranial hemorrhage and cerebrovascular disease^20^, suggesting that ZIP8 may play a role in the BBB.

Despite the strong genetic association between ZIP8^A391T^ and schizophrenia, the role of ZIP8 and its variants in schizophrenia have remained largely uncharacterized. Here, we report that the ZIP8^A391T^ mutation is a loss-of-function allele that leads to changes in glutamate receptor expression and function, likely through decreased zinc import at the synapse. We further provide evidence that replacing the ZIP8^A391T^ mutation with wild-type ZIP8 (ZIP8^WT^) or removal of the excess zinc at the synapse resolves these deficits. In addition, we show that excessive zinc results in elevated neuroinflammatory responses both in vitro and in vivo. As emerging evidence strengthens the link between neuroinflammation and glutamate hypofunction in psychiatric disorders^21,22^, this study provides a potential disease mechanism of schizophrenia that includes both of these phenomena, suggesting a novel direction for developing future therapeutics targeting schizophrenia.

## Materials and methods

All experiments involving animals in this study were carried out in accordance with the recommendations of the Guide for the Care and Use of Laboratory Animals. The protocol was approved by the Pfizer Institutional Animal Care and Use Committee.

### DNA constructs and antibodies

Constructs overexpressing human *ZIP8*^*WT*^, *ZIP8*^*A391T*^, *ZIP8*^*WT*^*-mCherry*, or *ZIP8*^*A391T*^*-mCherry* and all lentiviral vectors expressing *Luc-Gfp*-shRNA or *Zip8-Gfp*-shRNA were synthesized by Genscript, Inc. Plasmid DNA was transfected into Chinese hamster ovary (CHO) cells or primary pyramidal neurons by Lipofectamine 3000 or Lipofectamine LTX, respectively (Thermo Fisher Scientific Inc.) according to the manufacturer’s protocol. Primary antibodies include rabbit anti-GluA1 (PC246, Millipore), rabbit anti-GluN1(AGC-001, Alomone labs), rabbit anti-GluN2A (AGC-002, Alomone labs), rabbit anti-GluN2B (AGC-003, Alomone labs), rabbit anti-GluA2/3 (AB1506, Millipore). Secondary antibody Alexa Fluor 568 was purchased from Thermo Fisher Scientific.

### Zinc import into CHO cells

**Fig. 1:**
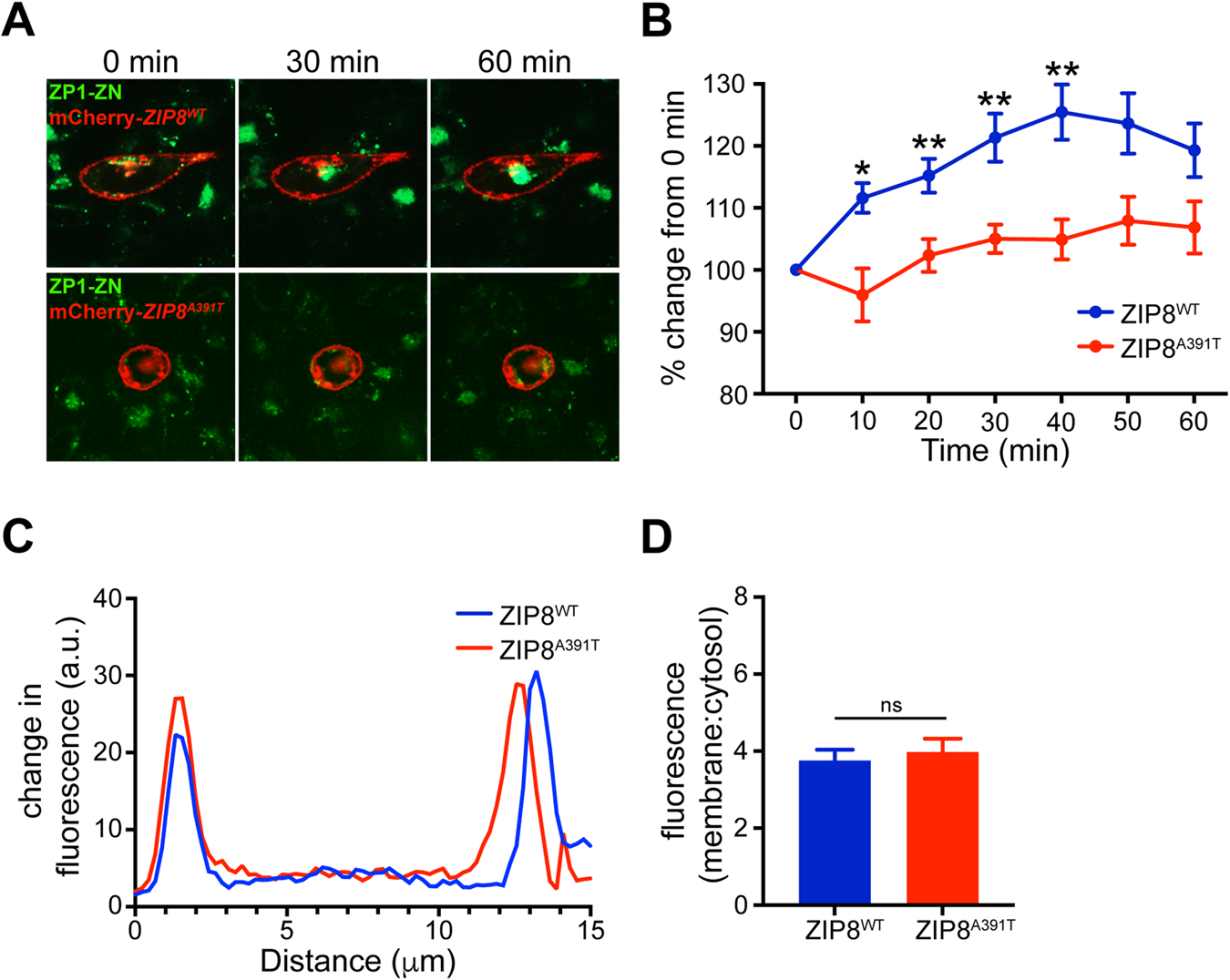
Human *ZIP8*^*A391T*^ SNP associated with schizophrenia is a loss-of-function allele. **A** Time course of ZP1 signal after addition of ZnCl_2_ in CHO cells overexpressing either *ZIP8*^*WT*^ or *ZIP8* with the human SNP mutation (*ZIP8*^*A391T*^) tagged with mCherry. **B** Quantification of GFP signals over time relative to 0 min for both ZIP8^WT^- and ZIP8^A391T^-overexpressing CHO cells. The baseline measurements (*T*0) of ZIP8^WT^ and ZIP8^A391T^ were not significantly different (unpaired *t*-test, *P* = 0.11). Compared to ZIP8^WT^, ZIP8^A391T^-expressing CHO cells had significantly lower ZnCl_2_ influx at multiple timepoints. *P* = 0.003 (two-way ANOVA, *n* = 25–32 per condition). Bonferroni’s post-hoc test revealed significant differences at 10, 20, 30, and 40 min (*P = 0.02, **P < 0.01). Error bars, SEM. **C**, **D** Overexpression of ZIP8^WT^ or ZIP8^A391T^ did not alter cell size (representative traces of cell size measurements shown in **C**). **D** Quantification of the membrane:cytosol signal. Ten cells from each group were used for quantification. Error bars, SEM.

The zinc import assay used in the present study was adapted from ref. ^23^. Briefly, CHO cells cultured on 6-well plates were transfected with either *ZIP8*^*WT*^*-mCherry* or *ZIP8*^*A391T*^*-mCherry* using Lipofectamine 3000 from Thermo Fisher Scientific. Two days before imaging, cells were plated onto 35 mm glass-bottom culture dishes containing 2 mL of growth medium. Cells were incubated in fresh growth medium with 5 µM ZP1 or DMSO and 4 µM Hoechst 33258 0.5 h before imaging. Cells were then washed twice with DMEM without phenol red, and 2 mL of serum-free DMEM containing 20 µM ZnCl_2_:pyrithione was applied at a ratio of 1:2. Cells overexpressing mCherry were imaged with live microscopy every 10 min for 60 min to monitor changes in green fluorescence signal. As no differences were observed between cell groups at the first imaging timepoint (*T*0), all other timepoints were normalized to each cell’s *T*0 to evaluate differences in ZnCl_2_ influx. Signal mean/standard deviation of each timeframe was analyzed using ImageJ. In determining the membrane targeting of *ZIP8*^*WT*^*-mCherry* or *ZIP8*^*A391T*^*-mCherry* construct, the cell expressing either construct was profiled using ImageJ to extract fluorescence signal across the cell while avoiding intracellular organelles such as nucleus and Golgi. The ratio of membrane/cytosol signal was quantified from ten cells per condition, and one representative trace of each condition is shown in Fig. 1C.

### Immunofluorescence staining

Rat pyramidal neurons were isolated and cultured on cover slips and then transfected with lentivirus encoding either *Luc*-*Gfp*-shRNA or *Zip8-Gfp-*shRNA at DIV7. At DIV28–30, cover slips were washed twice with conditioned culture media (Neurobasal, B27, and Glutamax) before the incubation with 100 µM ZX1 at 37 °C for 0, 30, or 180 min. During the last 30 min of incubation, primary antibodies against different glutamate receptor subunits were added to the cover slip at 37 °C for 30 min. All cover slips were then washed three times with conditioned culture media and incubated with secondary Alexa fluor 568 antibody 1:100 (Life Technologies) at 37 °C for 30 min. Cover slips were then washed three times with PBS, fixed with 4% paraformaldehyde/4% sucrose at room temperature for 15 min, and then mounted with ProLong Gold solution (Thermofisher). Confocal microscopy was performed using a Zeiss inverted microscope. All images were analyzed and quantified using custom Matlab scripts (code available upon request).

### Patch clamp of rat cortical culture

Rat fetal (E18) frontal cortex primary neurons were grown on 12-mm round poly-D-lysine-coated cover slips. Neurons were transfected with shRNA with or without rescue *ZIP8* plasmids using Lipofectamine 3000 (Thermo Fisher) at DIV7 following the manufacturer’s protocol. At DIV28–30, cover slips with primary neurons were used for patch clamp studies investigating α-amino-3-hydroxy-5-methyl-4-isoxazolepropionic acid receptor (AMPAR)- and NMDAR-mediated glutamatergic spontaneous activity and miniature excitatory postsynaptic currents (mEPSCs) as described previously^24^. In brief, 100 µM bicuculline was added to the external solution to block γ-aminobutyric acid (GABA)_A_ receptors throughout the recording. After break-in, 2 min of activity was recorded until a stable state was reached. (2R)-amino-5-phosphonovaleric acid (APV) (50 µM) was then applied for 5–6 min to block NMDARs and isolate AMPA EPSCs, followed by wash out from external buffer with bicuculline. Conversely, 10 µM 6-cyano-7-nitroquinoxaline-2,3-dione (CNQX) was then applied for another 5–6 min to block AMPARs and isolate NMDA EPSCs, followed by the application of both APV and CNQX to get baseline activity (Fig. S1D). For mEPSC recordings, 1 µM tetrodotoxin (TTX) was added to the external solution to block neuronal firing in addition to the sequence of APV and CNQX as above to acquire AMPA- and NMDA-mediated mEPSCs, respectively. Baseline activity of mEPSCs was obtained by the application of both APV and CNQX at the end of each recording (Fig. S1E). All mEPSC recordings were analyzed by Mini Analysis Program (Synaptosoft). Detection parameters were set as follows: threshold at 1, period to search a local maximal at 80 ms, time before a peak for baseline at 20 ms, period to search a decay at 40 ms, fraction of peak to find a decay time at 0.37, period to average a baseline at 10 ms, and area threshold at 2. Cells were rejected from analysis if the series resistance was greater than 15 MΩ after membrane rupture. Each event was then examined manually to ensure the accuracy of detection.

### Mice surgery and slice electrophysiology

CA1 neuronal cell bodies were labeled by bilateral stereotaxic injection of 6–8-week-old male C57/B6 mice with lentiviral vectors encoding either *Luc*-*Gfp*-shRNA or *Zip8-Gfp-*shRNA at the ventral hippocampus (bregma −3.4 mm; *X* ± 3.35 mm; *Z* −3.8 and −3.4 mm, 0.5 µL/z). Lentiviral vectors were purchased from Origene. Mice surgeries, the preparation of acute brain slices, and the setup of the recording system were conducted according to a previously published study^25^. The detailed protocol for Schaffer collateral stimulation was performed as previously reported^26^. Patch pipettes with 3–5 MΩ resistances were filled with internal solution. Cells were rejected from analysis if the series resistance was greater than 30 MΩ after membrane rupture.

### Biotinylation and Western blot

Rat pyramidal neurons were isolated and cultured on 6-well dishes and then transfected with lentivirus encoding either *Luc*-*Gfp*-shRNA or *Zip8-Gfp-*shRNA at DIV7. At DIV28–30, biotinylated lysates were prepared and blotted according to a published protocol^27^. After primary antibody incubation, blots were developed using a Typhoon imaging system (GE Healthcare) and the band intensity was quantified using ImageJ.

### Lipopolysaccharide challenge and measurement of cytokines

Male B6:129S *Zip8*^*+/−*^ mice and age-matched wild-type mice (6–17 weeks) were given a first 2.5 mg/kg dose of lipopolysaccharides (LPS) by intraperitoneal injection, followed by a second injection 6 h later, and a third injection 24 h after the first injection. An intravenous retro-orbital injection of 0.1 mL 70 kDa dextran-488 (Thermo Fisher) was given 28 h after the first LPS injection. After 30 min, each animal was euthanized, and the brain was collected and bisected for biochemistry and histopathological analysis. Blood (5 mL) was drawn and serum was isolated and tested in Meso Scale Discovery assays according to the manufacturer’s protocol to analyze the levels of various cytokines. Brain homogenate was prepared by adding 5 volumes of 20 mM Tris-HCl buffer (pH 7.6) with a protease inhibitor cocktail and sonicating with a probe. FITC signal from the dextran injection was measured using a spectrometer and normalized by total protein concentration determined by BCA analysis.

### NFκB activation by TNFα stimulation

HEK293 cells stably expressing a 3xNFκB reporter coupled to luciferase were generated in house. The day before transfection, HEK-NFκB cells were seeded onto 6-well plate at 500,000 cells per well. The next day, 2 µg of plasmids encoding different *ZIP8* transcripts were transfected into HEK-NFκB cells using Lipofectamine 2000 following the manufacturer’s protocol with a 3:1 ratio of lipid to DNA. After 24 h, the cells were re-seeded onto poly-D-lysine-coated clear-bottom 96-well plates at 50,000 cells per well. On the following day, the growth medium was replaced with 50 µL of medium containing various amounts of TNFα (1, 3, 10, 30, 100, or 300 ng/μL). After 6 h of incubation, 50 µL of 2× One Glo (Promega) was prepared and added to each well. The plate was incubated in the dark for 10 min prior to the readout of luminescence using Envision (Perkin Elmer).

## Results

### The *ZIP8*^*A391T*^ SNP associated with schizophrenia patients in GWAS studies is a loss-of-function allele

To determine the nature of the human SNP rs13107325 variant encoding an A391T change in ZIP8, we generated human *ZIP8*^*WT*^ and *ZIP8*^*A391T*^ cDNA constructs tagged with mCherry and overexpressed them in CHO cells, a commonly used cell line for metal ion transport studies. Similar membrane localization was observed in cells overexpressing either *ZIP8*^*WT*^*-mCherry* or *ZIP8*^*A391T*^*-mCherry* construct, indicating that A391T mutation did not alter the membrane targeting of ZIP8 (Fig. 1). Cells were loaded with ZP1 green fluorescing dye^23^ and live-imaged following the addition of ZnCl_2_ ions to the medium. The observation of increased green fluorescence signal into the cells was an expected result from overexpression of a functional ZIP8 transporter. In CHO cells overexpressing *ZIP8*^*WT*^*-mCherry*, we observed the expected time-dependent increase (25% at 40 min) of green fluorescence signal (Fig. 1A, B), indicating the robust import of ZnCl_2_ into the cytosol where it conjugates with ZP1 dye and emits green fluorescence. Cells overexpressing *ZIP8*^*A391T*^*-mCherry*, however, had a much slower and smaller increase (5% at 60 min) of the ZP1–ZnCl_2_ fluorescence signal, suggesting an impairment of ZnCl_2_ import as a result of the A391T mutation. Importantly, we did not observe any differences in cell size or membrane targeting of ZIP8^WT^ or ZIP8^A391T^ (Fig. 1D, C). These data indicate that the SNP rs13107325 is a loss-of-function mutation in the ZIP8 transporter.

**Fig. 2:**
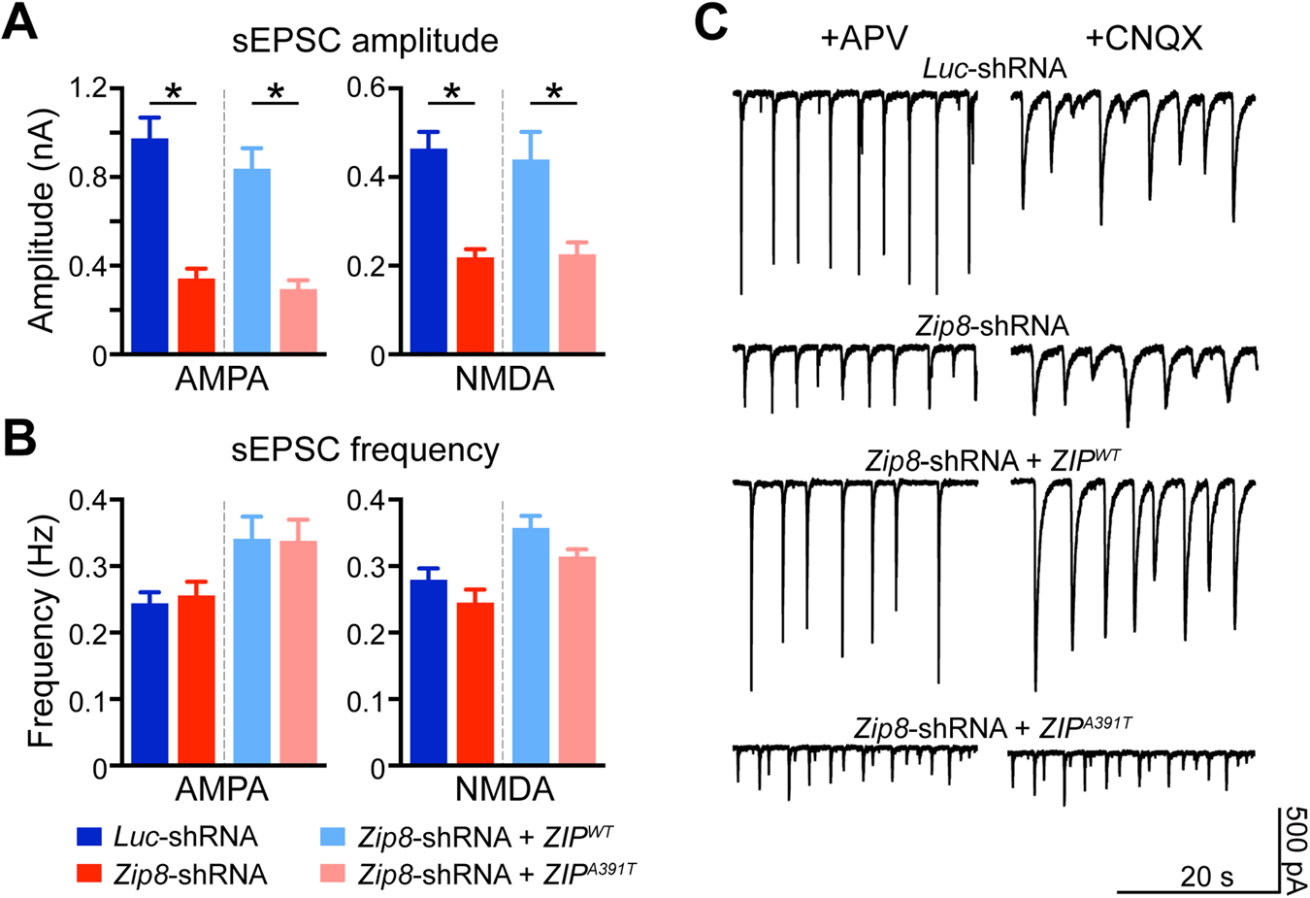
ZIP8 total knockout (KO) and loss-of-function allele A391T displays neuronal activity-dependent deficits in glutamate receptor-mediated sEPSCs. **A**, **B** Quantification of sEPSC amplitude (**A**) and frequency (**B**) recorded from cultured D28-30 neurons. *P < 0.05 (Student’s *t*-test, *n* = 6–10 per group). Error bars, SEM. **C** Representative raw traces of glutamate receptor-mediated sEPSCs in cultured pyramidal neurons expressing either *Luc*-shRNA, *Zip8*-shRNA, *Zip8*-shRNA + *ZIP8*^*WT*^ plasmid, or *Zip8-*shRNA + *ZIP8*^*A391T*^ plasmid. Note the significant loss of sEPSC amplitude, not frequency, in the presence of the ZIP8^A391T^ mutation, consistent with the loss-of-function observed in Fig. 1. No changes were observed in mEPSC amplitude or frequency (Fig. S1).

Zinc is known to be an allosteric inhibitor of NMDA receptors^16^. Given the evidence for glutamate hypofunction as a major pathology in schizophrenia and that glutamate receptors are inhibited by excess zinc at axon terminals upon neuronal activity, we addressed the role of zinc import by ZIP8 in neurotransmission by using short hairpin RNA (shRNA) lentivirus targeting *Zip8* or Luciferase in cultured rat hippocampal neurons. Neurons transfected with a hairpin targeting *Zip8* 3 weeks prior to recording (*Zip8*-shRNA) resulted in 90% knockdown (KD) of *Zip8* mRNA expression. Whole-cell patch clamp revealed significantly smaller amplitudes of both AMPAR- and NMDAR-mediated spontaneous excitatory postsynaptic currents (sEPSCs) in *Zip8*-shRNA neurons compared to *Luc*-shRNA transfected controls (Fig. 2A, C). The reduction of sEPSC amplitude in cultured neurons was rescued by co-expressing *ZIP8*^*WT*^ but not *ZIP8*^*A391T*^ construct (Fig. 2A, C), consistent with our finding that *ZIP8*^*A391T*^ is a loss-of-function allele that disrupts the import of synaptic zinc. sEPSC frequency was not affected by either *ZIP8*^*WT*^ or *ZIP8*^*A391T*^ transfection (Fig. 2B). These results support the hypothesis that ZIP8 hypofunction may lead to decreased zinc import and thus increased synaptic zinc and zinc-mediated AMPAR and NMDAR inhibition.

To ensure we were observing solely a postsynaptic effect, we next applied TTX to eliminate major neuronal activity in the culture and recorded mEPSCs caused by quantal release of presynaptic vesicles independent of stimulation in presynaptic termini. Interestingly, we did not observe a difference between ZIP8 KD and rescued neurons in terms of either mEPSC amplitude or frequency (Fig. S1A–C), suggesting that the deficit in sEPSCs recorded without TTX is activity dependent. Given that zinc can be co-released with glutamate at places such as hippocampal mossy fiber synapses^28^, it is likely that only during a firing event could zinc concentration rise to the levels required for inhibition of postsynaptic glutamate receptors^16^ in the synaptic cleft.

### ZIP8 knockdown leads to decreased surface localization of glutamate receptor subunits

**Fig. 3:**
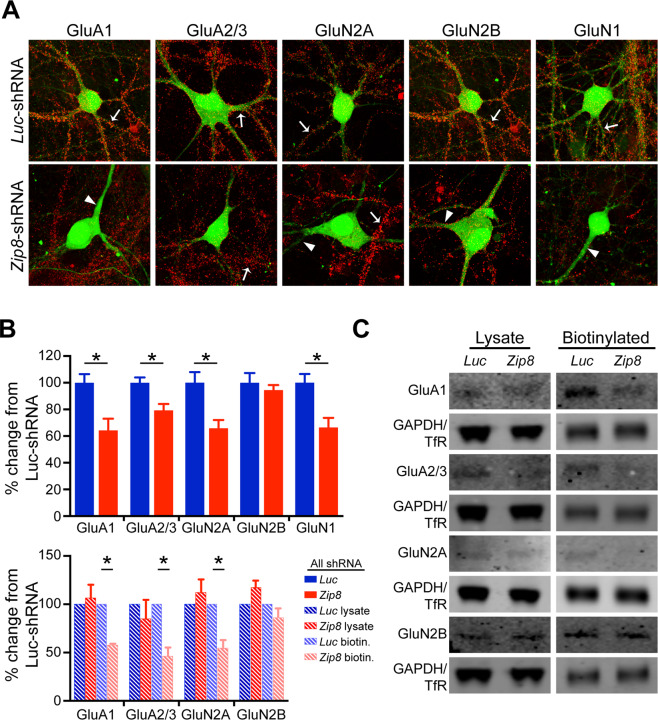
ZIP8 KO in cultured neurons leads to decreased surface localization of specific glutamate receptor subunits. **A** Representative neurons expressing GFP-tagged *Luc-* or *Zip8*-shRNA and stained with antibodies against different glutamate receptor subunits at DIV28–30. Arrows denote neurites from *Luc*-shRNA-expressing neurons (top) or surrounding non-transfected neurons in the *Zip8*-shRNA condition (bottom). Arrow heads indicate neurites from *Zip8*-shRNA-expressing neurons. **B** Quantification of different glutamate receptor subunits’ staining intensity relative to *Luc*-shRNA treatment. Intensity of signal for each subunit from the *Luc*-shRNA treatment was averaged and used to normalize *Luc*- and *Zip8*-shRNA treatments. *P < 0.05 (Student’s *t*-test); *n* = 6–10 per group. Error bar, SEM. **C** Representative western blot of individual glutamate receptor subunits, as quantified in **B**.

To evaluate whether the reduced amplitude of glutamate receptor-mediated sEPSCs is due to decreased surface localization of postsynaptic glutamate receptors, we utilized live labeling of common AMPA and NMDA glutamate receptor subunits and analyzed the receptor surface population. In cultured hippocampal neurons transfected with shRNA targeting *Zip8*, we found a significant reduction of surface glutamate receptor subunits, including GluA1, GluA2/3, GluN1, and GluN2A (Fig. 3A, B). Consistent with the finding that zinc has a 50-fold lower affinity for GluN2B-containing NMDARs^13,29–31^, we did not observe a change in the surface expression of this receptor subtype (Fig. 3A, B).

To confirm our initial findings that knockdown of ZIP8 leads to increased zinc levels and decreased glutamate receptor surface expression, we used a surface biotinylation assay to query the levels of surface versus total glutamate receptor expression under different conditions. Only the biotinylated populations of certain glutamate receptor subunits, including GluA1, GluA2/3, and GluN2A, were decreased in ZIP8 KD neuronal lysates (Fig. 3). Consistent with the live labeling experiments, the GluN2B subunit did not show significant changes in biotinylated levels in ZIP8 KD lysates (Fig. 3C). Importantly, all glutamate receptor subunits had similar levels of total protein amounts between *Luc*-shRNA- and *Zip8*-shRNA-treated neurons, demonstrating that the surface levels of specific glutamate receptor subunits that are susceptible to synaptic zinc inhibition underwent dynamic activity-dependent regulation.

### Chelation of extracellular zinc rescues glutamate receptor surface localization and function in ZIP8 knockdown neurons

**Fig. 4:**
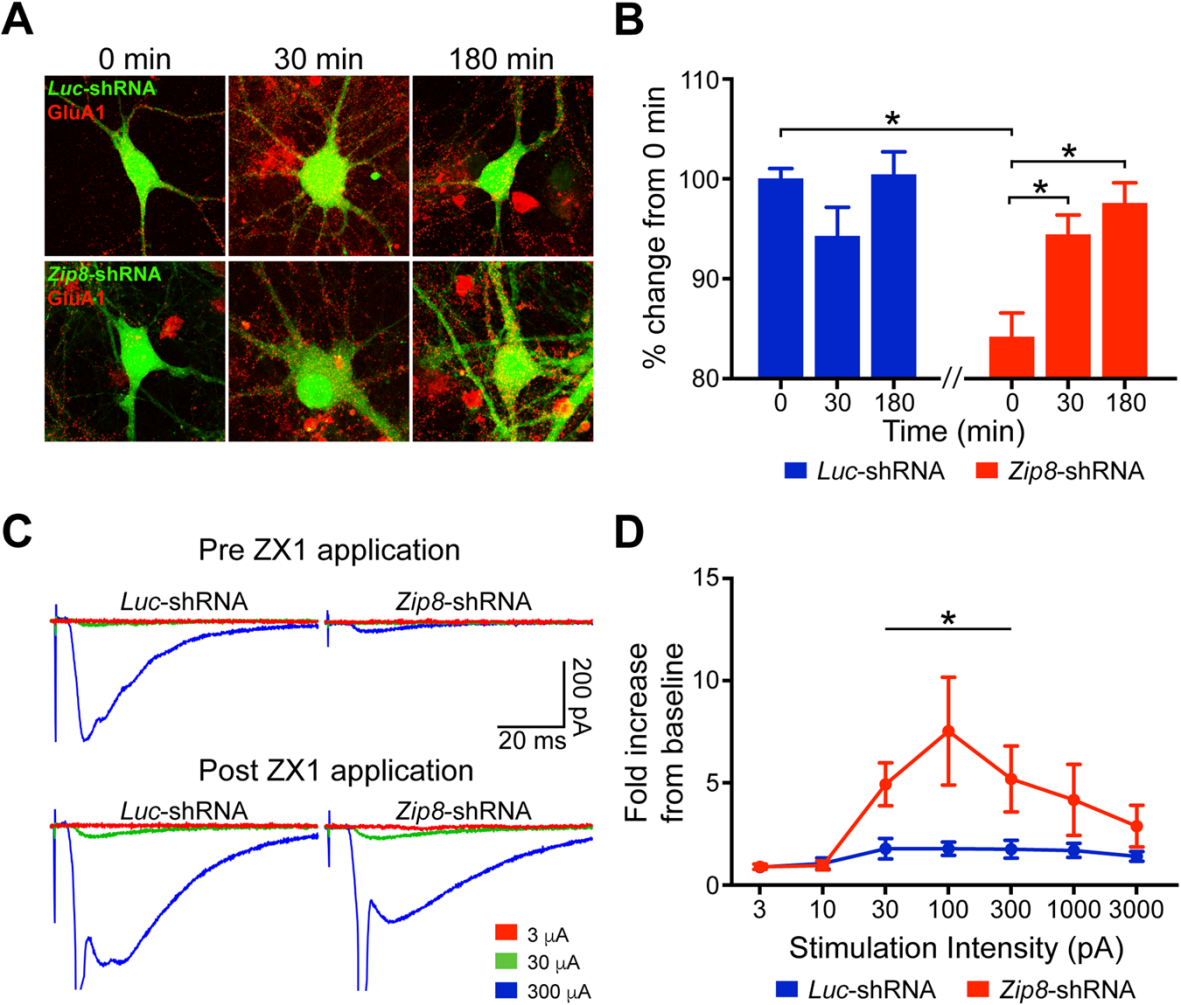
Zinc chelation rescues GluA1 receptor surface localization and evoked EPSC amplitude in hippocampal CA1 neurons. **A** Time course of 100 μM ZX1 treatment in *Luc*- or *Zip8*-shRNA-expressing cultured pyramidal neurons stained with antibodies against the GluA1 subunit. **B** Quantification of GluA1 puncta over time following ZX1 application. Data are normalized to *Luc*-shRNA at 0 min. Note the significant difference between treatments prior to zinc chelation (two-way ANOVA followed by Sidak’s post hoc test; *Luc* vs. *Zip8* at 0 min (*P* < 0.001), *Zip8* 0 min vs. 30 min (*P* < 0.02), *Zip8* 0 min vs. 180 min (*P* < 0.04); *n* = 6–10 per group). Error bar, SEM. **C** Representative traces of Schaffer collateral-evoked EPSCs from hippocampal CA1 neurons transfected in vivo with virus encoding either *Luc*- or *Zip8*-shRNA then recorded ex vivo using whole-cell patch clamp. Recordings made from Schaffer collateral stimulation before and after treatment of ZX1 (*n* = 6 per group). Note that the same stimulation intensities given prior to ZX1 now result in a large population event, typical of a loss of zinc-mediated inhibition^33,34^. **D** Quantification of evoked signals at different stimulation intensities shown to the right (two-way ANOVA followed by Sidak’s post hoc test; *Luc* + ZX1 vs. *Zip8* + ZX1 effect of treatment (*P* < 0.0001)). Error bars, SEM.

Given our results suggesting that ZIP8 loss-of-function may lead to the accumulation of free zinc in the synaptic cleft and the subsequent inhibition of glutamate receptors, we next evaluated whether removing the excess zinc would reverse the phenotypes observed in ZIP8 KD neurons. We focused our analyses on GluA1 receptor expression because of its robust expression and thus larger dynamic window for immunocytochemistry. Using ZX1, a potent extracellular zinc chelator impermeable to neurons^32^, we observed a restoration of GluA1 subunits on the surface of ZIP8 KD cells after 30 min of incubation with ZX1, which persisted up through 180 min of ZX1 preincubation (Fig. 4A, B). This result further suggests that the decreased surface levels of certain glutamate receptor subunits are reversible, as would be expected from the activity-dependent dynamic regulation of receptor surface levels (Figs. 2A, C and S1).

We next examined whether our observations in cultured neurons would translate to the intact brain. One month after injecting lentivirus encoding either *Luc-Gfp*-shRNA or *Zip8-Gfp-*shRNA into the hippocampal CA1 region of C57/B6 mice, we recorded the Schaffer collateral-evoked glutamate-mediated excitatory activity from GFP-labeled hippocampal CA1 pyramidal cells. Prior to application of ZX1, control *Luc*-shRNA-treated CA1 neurons showed higher evoked glutamate receptor activity compared to *Zip8*-shRNA-treated neurons (Fig. 4C, D), consistent with our results in cultured neurons (Fig. 2). In agreement with the rescue of glutamate receptor surface expression (Fig. 4A), evoked currents in CA1 neurons infected with virus encoding *Zip8*-shRNA were normalized compared to control neurons (Fig. 4C, D) 30 min after ZX1 application, indicative of removal of all zinc-mediated inhibition^33,34^. Together with our immunofluorescence data, these data are consistent with the suggestion that ZIP8 loss-of-function leads to an accumulation of zinc that inhibits synaptic glutamate receptors, which can be reversed by removal of extracellular zinc. These results suggest that ZIP8 is, at least in part, responsible for the dynamic regulation of synaptic glutamate-mediated signaling.

### ZIP8 haploinsufficiency leads to an elevated innate immune response in the central nervous system

**Fig. 5:**
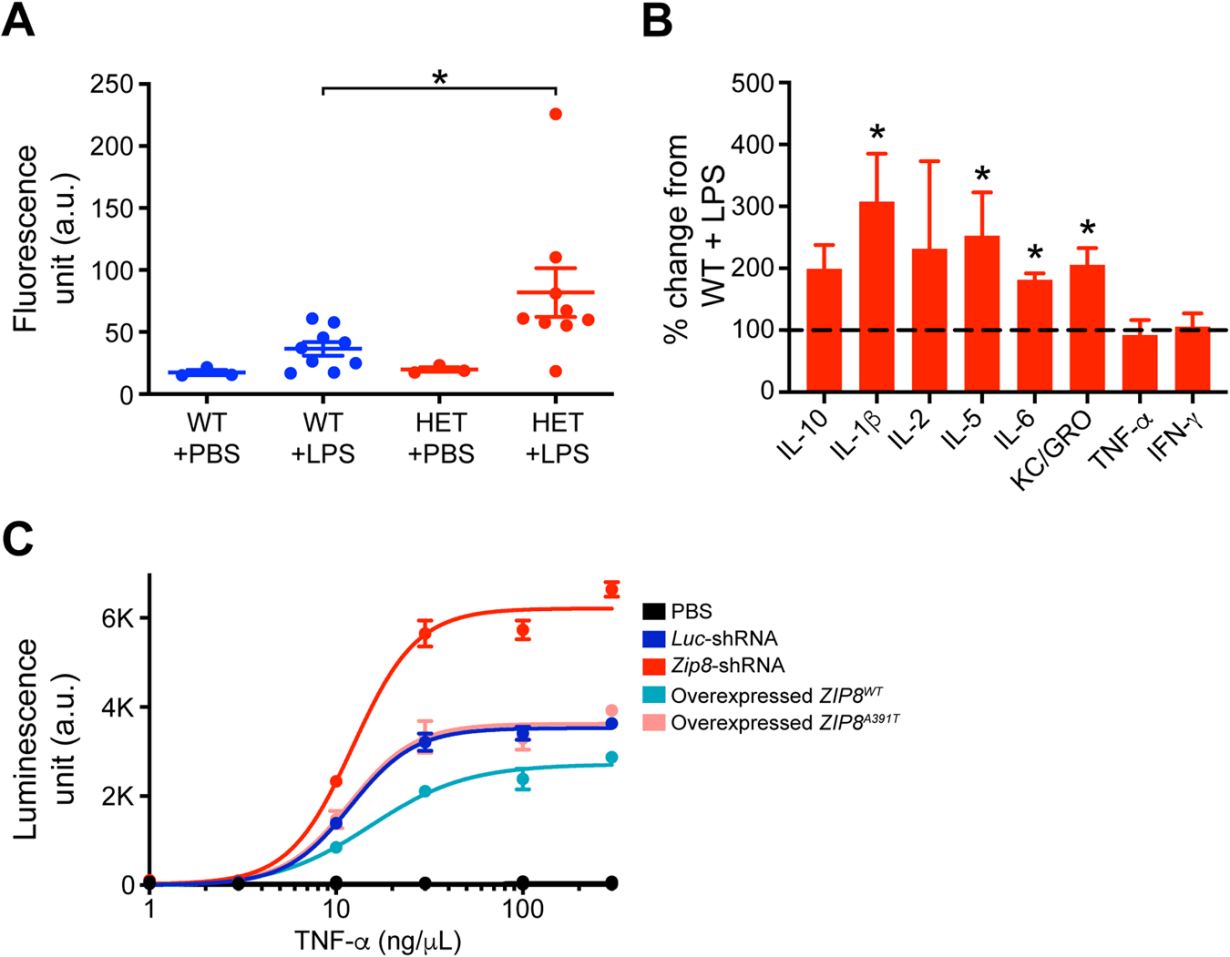
ZIP8 loss-of-function results in increased BBB leakage and overactivation of NFκB. **A** Intravenous retro-orbital sinus injection of Dextran-488 after 24 h LPS treatment. Mice that were haplo-insufficient for *Zip8* demonstrated an elevated dextran 488 level compared to WT mice. *P < 0.05 (Student’s *t*-test; *n* = 3 per PBS-treated group and *n* = 7–10 per LPS-treated group). Error bar, SEM. **B** *Zip8* HET mice after 24 h LPS treatment displayed increased levels of several plasma cytokines compared to WT mice (one-way ANOVA followed by Tukey’s multiple comparison test, * = significant difference between WT LPS and HET LPS conditions: KC/GRO, *P* = 0.042; IL-5, *P* = 0.05; IL-1β, *P* = 0.05; IL-6, *P* = 0.002; see Fig. S2). Cytokine levels from WT mice treated with LPS were averaged and used for normalizing cytokine response from *Zip8* HET mice treated with LPS (*n* = 17 per group). Error bar, SEM. **C** In HEK293 cells stably expressing a 3xNFκB reporter, *Zip8*-shRNA transient transfection led to increased NFκB activation compared to *Luc*-shRNA transiently transfected cells, whereas cells transfected with *ZIP8*^*WT*^ plasmid displayed a protective effect compared to cells transfected with *ZIP8*^*A391T*^ plasmid, which had no effect on the WT background (two-way ANOVA followed by Bonferonni’s post hoc test; TNFa vs. manipulation interaction (*P* < 0.0001). See Table S1 for details. Error bar, SEM.

While our characterization of the loss-of-function A391T allele suggests that ZIP8 plays a role in regulating glutamate receptor function, it is likely that ZIP8 haploinsufficiency has other deleterious effects, especially given its previously reported role in inflammation. ZIP8 has been reported to affect innate immune functions through coordination of zinc metabolism and inhibition of NFκB^35^. Interestingly, RNAseq data from Barres and colleagues showed highly enriched ZIP8 expression in brain endothelial cells compared with endothelial cells in peripheral systems^36^, suggesting additional roles of ZIP8 in mediating the immune response in places such as the BBB. Given the evidence that complete KO of *Zip8* in mice is neonatally lethal with severe developmental deficits in growth, organ morphogenesis, and hematopoiesis^37^, we obtained ZIP8 haploinsufficient mice (*Zip8*^+/−^) (courtesy of Dr. Daniel J. Rader, University of Pennsylvania) to investigate the role of ZIP8 in the central nervous system (CNS). We injected *Zip8*^+/−^ mice or control littermates with fluorescent-labeled dextran to examine the integrity of the BBB after stimulation of the innate immune response with a high dose of LPS. Following LPS injections, we detected significantly higher dextran-488 signals in the brain lysates of *Zip8*^*+/−*^ mice compared to control littermates (Fig. 5A), indicating that ZIP8 hypomorphic animals displayed a leakier BBB upon inflammation. Using meso-scale discovery assays for various cytokine levels, we then confirmed that several key cytokines, including IL-6 and IL-1β, were elevated in the plasma of *Zip8*^*+/−*^ mice compared to control littermates (Figs. 5B and S2). Furthermore, using HEK293 cells stably expressing a luciferase NFκB reporter cassette, we found that shRNA-mediated knockdown of ZIP8 led to higher NFκB activation upon TNFα stimulation as compared to *Luc*-shRNA transfected cells (Fig. 5C and Table S1). Conversely, overexpression of the *ZIP8*^*WT*^ construct decreased NFκB activation compared to the wild-type background, whereas the overexpression of *ZIP8*^*A391T*^ did not (Fig. 5C and Table S1). Together, these results demonstrate that ZIP8 deficiency leads to morphological changes at the BBB and potentially harmful hyperactivation of inflammatory pathways.

## Discussion

Although GWAS studies have yielded insights into the genetic architecture underlying risk for schizophrenia, a molecular understanding of how each perturbation translates into the pathology of the disease is still lacking. In this study, we elucidated the biological significance of a human SNP rs13107325 as a loss-of-function A391T allele in zinc transporter ZIP8. We demonstrated that this loss-of-function leads to glutamate receptor hypofunction that is at least in part due to downregulated surface localization of key synaptic glutamate receptors. Finally, we showed that the ZIP8^A391T^ mutation results in an elevated innate immune response. These findings are consistent with observations from patients with schizophrenia and support the glutamate dysfunction^38^ and immune dysfunction^39^ observed in numerous studies. To the best of our knowledge, our data represent the first characterization of the functional consequence of the A391T allele of *ZIP8* in CNS glutamate function and are consistent with immune dysfunction observed with the ZIP8^A391T^ mutation in Crohn’s disease^40^.

Taken together, our results align with the glutamate hypofunction hypothesis, a central concept of schizophrenia. This theory arises from observations that (1) a majority of the genes that are associated with an increased risk for schizophrenia can influence the function of NMDARs or related receptor-interacting proteins and signal transduction pathways^41,42^; (2) subjects with schizophrenia show profound deficits in neural signals such as mismatch negativity (MMN), thought to be NMDA dependent^43^ and predictive of conversion from the prodromal state^44^; (3) NMDAR antagonists such as phencyclidine and ketamine produce deficits in MMN and cognition as well as psychomimetic effects in healthy individuals reminiscent of symptoms of schizophrenia^45,46^; and (4) mice with reduced NMDAR expression display behaviors thought to be a reflection of psychomimetic effects, such as increased locomotor activity and rearing^47^. Although there was no previous link between NMDARs and the zinc transporter ZIP8, it is well known that NMDARs, particularly the GluN2A subunit, are highly sensitive to synaptic zinc^13,16^. Once ZIP8 is downregulated in neurons, as in our study with hairpin or with expression of the loss-of-function *ZIP8*^*A391T*^ allele, zinc import into the cell mediated by ZIP8 is impaired, and zinc would thus have the opportunity to accumulate in the synaptic cleft. Our results predict that individuals carrying the *ZIP8*^*A391T*^ allele would have decreased zinc import and consequently elevated levels of synaptic zinc, ultimately leading to glutamate hypofunction due to increased inhibition and/or decreased surface expression of GluN2A-containing NMDARs and AMPARs. Future studies should focus on the complexities of zinc dynamics in the presence of the *ZIP8*^*A391T*^ allele.

Our findings that neuronal KD of ZIP8 as well as replacement with ZIP8^A391T^ results in decreased amplitude of NMDA- and AMPA-mediated sEPSCs likely represent an increase in synaptic zinc due to a decrease in the control of synaptic zinc. This probable increase of zinc in the extrasynaptic space likely dampens NMDAR- and AMPAR-mediated activity, leaving a modest amount of postsynaptic activity intact as demonstrated in our electrophysiological data (Fig. 2). Notably, the reduction of GluN2A, but not GluN2B, surface expression is consistent with data showing that zinc is many-fold more selective for GluN2A receptors^13,29–31,48,49^ and AMPARs^34^. We found that persistent, but not total, inhibition of these receptors by dysregulation of synaptic zinc leads to receptor internalization of GluN2A-NMDARs^50^ and GluA1-AMPARs as suggested by decreased surface expression (Fig. 3), perhaps mimicking a form of long-term depression (LTD)^51^. Our finding that surface expression of GluN2B-containing receptors was not affected by ZIP8 KD or the ZIP8^A391T^ mutation is likely because the IC_50_ of zinc for these receptor subtypes is at least 50-fold higher compared to GluN2A-NMDARs^13,48,49^. Prior findings have demonstrated that GluN2B-, but not GluN2A-, containing NMDARs are required for induction of LTD^52,53^. Therefore, the increased inhibition selectively at GluN2A-containing NMDARs, leaving GluN2B-mediated NMDA signaling intact, may explain the decrease in GluA1 receptor surface expression observed (Fig. 3), similar to that observed when GluN2A-containing NMDARs are knocked down^54^.

The demonstration that overexpression of *ZIP8*^*WT*^ and the chelation of extracellular zinc rescues both receptor surface expression (Fig. 4A–D) as well as function (Figs. 2 and 4) further supports the hypothesis that the ZIP8^A391T^ mutation results in glutamate hypofunction consistent with that observed in schizophrenia. Interestingly, the chelation of zinc over a 30-min period not only rescued evoked EPSCs but exacerbated the population spike amplitude compared to the luciferase condition. This finding is unsurprising, however, as chelation of zinc with ZX1 results in loss of all endogenous zinc content, thus removing any ongoing zinc-mediated inhibition. These data are consistent with the increase in LTP observed following chelation of zinc in hippocampal slices^55^.

Finally, chronic inflammation in the CNS is considered pathogenic and hypothesized to be responsible for many diseases such as multiple sclerosis and schizophrenia^56,57^. In schizophrenia, dysregulation of the immune system is often evidenced in the elevation of type-2 cytokines such as IL-6 and IL-10 (ref. ^58^). Although zinc is thought to be important in the peripheral innate immune system as an inhibitor of NFκB^35^, little is known about the role of either zinc or ZIP8 in regulating immune responses in the CNS. In our study, *Zip8* hypomorphic mice displayed deficits in the integrity of the BBB after LPS challenge as shown by a greater dye concentration collected in the brain homogenate following intravenous injection compared to controls. This finding reflects a hallmark condition of CNS inflammation in which a compromised BBB facilitates transmigration of lymphocytes^59^. Blood samples from the *Zip8*^*+/−*^ mice, collected after LPS treatment, displayed higher levels of several cytokines, including IL-6 and IL-10, which have been shown to be elevated in schizophrenia. Our results also showed that overexpressing ZIP8^WT^ protein in NFκB reporter cell lines decreased NFκB signaling, whereas overexpressing the ZIP8^A391T^ variant did not. This result correlates well with previous observations that ZIP8 KO and A391T allele in cellular systems are linked to an elevated immune response^35,40,60^. Together, these findings implicate CNS inflammation as another avenue by which ZIP8 dysfunction could increase the risk for psychiatric disease. Whether immune system dysregulation or glutamate hypofunction is more central to the ZIP8^A391T^ mutation’s risk remains to be elucidated, and future work should be done to better understand the extent to which these different pathways might interact. Future studies should focus on full characterization of behavioral phenotypes in the ZIP8^A391T^ mouse before and after specific manipulation of either CNS activity or inflammation.

Given the reported CNS inflammation in schizophrenia patients^22,56^, ZIP8 hypofunction (as in the context of the A391T allele) could engender psychiatric risk both by leading to glutamate receptor hypofunction as well as increased inflammation. Our data indicate that targeted treatment with mechanisms selectively enhancing glutamate function and/or targeting anti-inflammatory mechanisms may benefit this subpopulation of patients with schizophrenia. Stratifying subjects based on their autoimmune response or using measures sensitive to NMDA-mediated responses to glutamate, such as MMN, may enable greater success for therapeutics aiming to treat schizophrenia.

## Supplementary information

Supplementary Material for Schizophrenia-associated SLC39A8 polymorphism is a loss-of-function allele altering glutamate receptor and innate immune signaling
