# Supplementary material for: Schizophrenia-associated SLC39A8 polymorphism is a loss-of-function allele altering glutamate receptor and innate immune signaling
Source: Transl Psychiatry. 2021 Feb 19;11:136. doi: 10.1038/s41398-021-01262-5 (PMC7895948; doi:10.1038/s41398-021-01262-5)
Supplement: Supplementary file 1 — Supplementary Material for Schizophrenia-associated SLC39A8 polymorphism is a loss-of-function allele altering glutamate receptor and innate immune signaling [file 41398_2021_1262_MOESM1_ESM.docx]

Supplementary Information for

**Schizophrenia-associated SLC39A8 polymorphism is a loss-of-function allele altering glutamate receptor and innate immune signaling**

Wei Chou Tseng^1^, Veronica R. Bieber^2^, Thomas A. Lanz^3^, Mark L. Weber^3^, Jincheng Pang^3^, Kevin X. Le^3^, Robert D. Bell^3^, Patricio O’Donnell^4^, and Derek L. Buhl^4^*

^1^Wave Life Sciences, Cambridge, MA 02138

^2^Biogen, Cambridge, MA 02139

^3^Pfizer, Inc., Cambridge, MA 02139

^4^Takeda Pharmaceuticals, Cambridge, MA 02139

* Corresponding author: Derek L. Buhl

**Email:**  derek.buhl@gmail.com

**This PDF file includes:**

Figure S1: mEPSC and baseline recordings complementing main Figure 2

Figure S2: Individual cytokine levels in plasma for data shown in Figure 5B

Table S1: Bonferonni’s post hoc test on data shown in Figure 5C


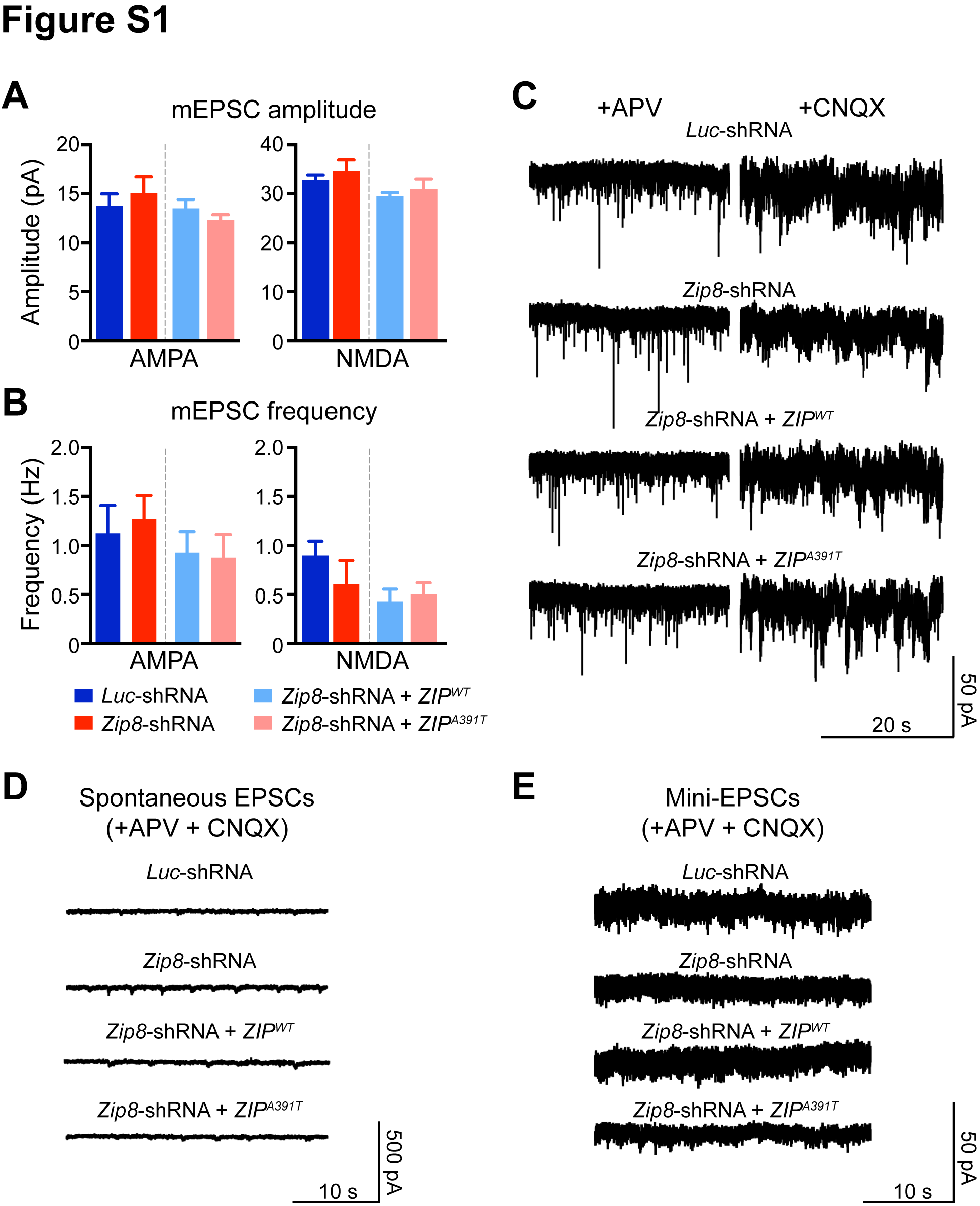


Fig. S1. ZIP8 total KO and loss-of-function allele A391T does not affect glutamate receptor-mediated mEPSCs. (A-B) Quantification of mEPSC amplitude (A) and frequency (B) recorded from cultured D28-30 neurons. (C) Representative raw traces of glutamate receptor-mediated mEPSCs in cultured pyramidal neurons expressing either *Luc*-shRNA, *Zip8*-shRNA, *Zip8*-shRNA + *ZIP8^WT^* plasmid, or *Zip8*-shRNA + *ZIP8^A391T^* plasmid. Unlike the effect observed on sEPSCs (Fig. 2), the presence of the ZIP8^A391T^ mutation does not have an effect on mEPSCs.


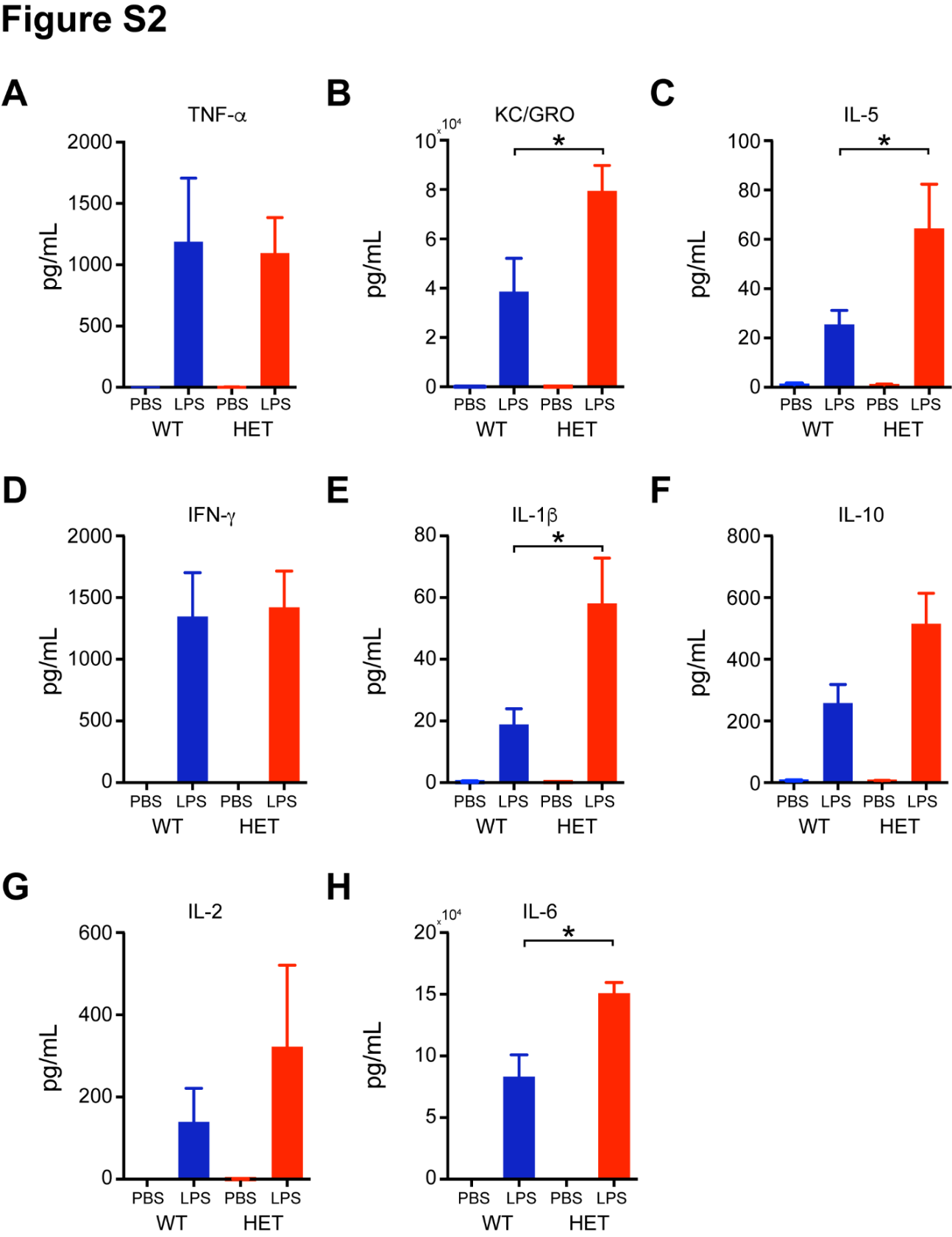


Fig. S2. *Zip8* HET mice after 24 hours LPS treatment displayed increased level of several plasma cytokines compared to WT mice. Breakdown comparison of several plasma cytokines shown in Fig. 5B. In response to LPS, *Zip8* HET mice showed a significant increase in numerous inflammatory markers (one way ANOVA followed by Tukey’s multiple comparison test, *= significant difference between WT LPS and HET LPS conditions: KC/GRO, P=0.042; IL-5, P=0.05; IL-1β, P=0.05; IL-6, P=0.002).

Table S1. Results of Bonferonni’s post hoc test on data shown in Figure 5C. Note that WT and KO represent *Luc*-shRNA and *Zip8*-shRNA, respectively.

| **TNFα (1 ng/μL)** | | | | | | | | |
| --- | --- | --- | --- | --- | --- | --- | --- | --- |
| **Contrast** | **Mean Diff.** | **95.00% CI of diff.** | | **Summary** | | | | **Adjusted P-Value** |
| WT+PBS - WT+TNFα | 0 | -483.8 to 483.8 | | | ns | | | >0.9999 |
| WT+PBS - KO+PBS | -20 | -503.8 to 463.8 | | | ns | | | >0.9999 |
| WT+PBS - KO+TNFα | -60 | -543.8 to 423.8 | | | ns | | | >0.9999 |
| WT+PBS - *ZIP8^WT^* + PBS | 20 | -463.8 to 503.8 | | | ns | | | >0.9999 |
| WT+PBS - *ZIP8^WT^* + TNFα | 10 | -473.8 to 493.8 | | | ns | | | >0.9999 |
| WT+PBS - *ZIP8^A391T^* + PBS | 0 | -483.8 to 483.8 | | | ns | | | >0.9999 |
| WT+PBS - *ZIP8^A391T^* + TNFα | -20 | -503.8 to 463.8 | | | ns | | | >0.9999 |
| WT+TNFα - KO+PBS | -20 | -503.8 to 463.8 | | | ns | | | >0.9999 |
| WT+TNFα - KO+TNFα | -60 | -543.8 to 423.8 | | | ns | | | >0.9999 |
| WT+TNFα - *ZIP8^WT^* + PBS | 20 | -463.8 to 503.8 | | | ns | | | >0.9999 |
| WT+TNFα - *ZIP8^WT^* + TNFα | 10 | -473.8 to 493.8 | | | ns | | | >0.9999 |
| WT+TNFα - *ZIP8^A391T^* + PBS | 0 | -483.8 to 483.8 | | | ns | | | >0.9999 |
| WT+TNFα - *ZIP8^A391T^* + TNFα | -20 | -503.8 to 463.8 | | | ns | | | >0.9999 |
| KO+PBS - KO+TNFα | -40 | -523.8 to 443.8 | | | ns | | | >0.9999 |
| KO+PBS - *ZIP8^WT^* + PBS | 40 | -443.8 to 523.8 | | | ns | | | >0.9999 |
| KO+PBS - *ZIP8^WT^* + TNFα | 30 | -453.8 to 513.8 | | | ns | | | >0.9999 |
| KO+PBS - *ZIP8^A391T^* + PBS | 20 | -463.8 to 503.8 | | | ns | | | >0.9999 |
| KO+PBS - *ZIP8^A391T^* + TNFα | 0 | -483.8 to 483.8 | | | ns | | | >0.9999 |
| KO+TNFα - *ZIP8^WT^* + PBS | 80 | -403.8 to 563.8 | | | ns | | | >0.9999 |
| KO+TNFα - *ZIP8^WT^* + TNFα | 70 | -413.8 to 553.8 | | | ns | | | >0.9999 |
| KO+TNFα - *ZIP8^A391T^* + PBS | 60 | -423.8 to 543.8 | | | ns | | | >0.9999 |
| KO+TNFα - *ZIP8^A391T^* + TNFα | 40 | -443.8 to 523.8 | | | ns | | | >0.9999 |
| ZIP8^WT^ + PBS - *ZIP8^WT^* + TNFα | -10 | -493.8 to 473.8 | | | ns | | | >0.9999 |
| ZIP8^WT^ + PBS - *ZIP8^A391T^* + PBS | -20 | -503.8 to 463.8 | | | ns | | | >0.9999 |
| ZIP8^WT^ + PBS - *ZIP8^A391T^* + TNFα | -40 | -523.8 to 443.8 | | | ns | | | >0.9999 |
| ZIP8^WT^ + TNFα - *ZIP8^A391T^* + PBS | -10 | -493.8 to 473.8 | | | ns | | | >0.9999 |
| ZIP8^WT^ + TNFα - *ZIP8^A391T^* + TNFα | -30 | -513.8 to 453.8 | | | ns | | | >0.9999 |
| ZIP8^A391T^ + PBS - *ZIP8^A391T^* + TNFα | -20 | -503.8 to 463.8 | | | ns | | | >0.9999 |
|  |  |  | | |  | | |  |
| **TNFα (3 ng/μL)** | | | | | | | | |
| **Contrast** | **Mean Diff.** | **95.00% CI of diff.** | **Summary** | | | | **Adjusted P Value** | |
| WT+PBS - WT+TNFα | -30 | -513.8 to 453.8 | | | | ns | >0.9999 | |
| WT+PBS - KO+PBS | -30 | -513.8 to 453.8 | | | | ns | >0.9999 | |
| WT+PBS - KO+TNFα | -70 | -553.8 to 413.8 | | | | ns | >0.9999 | |
| WT+PBS - *ZIP8^WT^* + PBS | -10 | -493.8 to 473.8 | | | | ns | >0.9999 | |
| WT+PBS - *ZIP8^WT^* + TNFα | -30 | -513.8 to 453.8 | | | | ns | >0.9999 | |
| WT+PBS - *ZIP8^A391T^* + PBS | 10 | -473.8 to 493.8 | | | | ns | >0.9999 | |
| WT+PBS - *ZIP8^A391T^* + TNFα | -70 | -553.8 to 413.8 | | | | ns | >0.9999 | |
| WT+TNFα - KO+PBS | 0 | -483.8 to 483.8 | | | | ns | >0.9999 | |
| WT+TNFα - KO+TNFα | -40 | -523.8 to 443.8 | | | | ns | >0.9999 | |
| WT+TNFα - *ZIP8^WT^* + PBS | 20 | -463.8 to 503.8 | | | | ns | >0.9999 | |
| WT+TNFα - *ZIP8^WT^* + TNFα | 0 | -483.8 to 483.8 | | | | ns | >0.9999 | |
| WT+TNFα - *ZIP8^A391T^* + PBS | 40 | -443.8 to 523.8 | | | | ns | >0.9999 | |
| WT+TNFα - *ZIP8^A391T^* + TNFα | -40 | -523.8 to 443.8 | | | | ns | >0.9999 | |
| KO+PBS - KO+TNFα | -40 | -523.8 to 443.8 | | | | ns | >0.9999 | |
| KO+PBS - *ZIP8^WT^* + PBS | 20 | -463.8 to 503.8 | | | | ns | >0.9999 | |
| KO+PBS - *ZIP8^WT^* + TNFα | 0 | -483.8 to 483.8 | | | | ns | >0.9999 | |
| KO+PBS - *ZIP8^A391T^* + PBS | 40 | -443.8 to 523.8 | | | | ns | >0.9999 | |
| KO+PBS - *ZIP8^A391T^* + TNFα | -40 | -523.8 to 443.8 | | | | ns | >0.9999 | |
| KO+TNFα - *ZIP8^WT^* + PBS | 60 | -423.8 to 543.8 | | | | ns | >0.9999 | |
| KO+TNFα - *ZIP8^WT^* + TNFα | 40 | -443.8 to 523.8 | | | | ns | >0.9999 | |
| KO+TNFα - *ZIP8^A391T^* + PBS | 80 | -403.8 to 563.8 | | | | ns | >0.9999 | |
| KO+TNFα - *ZIP8^A391T^* + TNFα | 0 | -483.8 to 483.8 | | | | ns | >0.9999 | |
| ZIP8^WT^ + PBS - *ZIP8^WT^* + TNFα | -20 | -503.8 to 463.8 | | | | ns | >0.9999 | |
| ZIP8^WT^ + PBS - *ZIP8^A391T^* + PBS | 20 | -463.8 to 503.8 | | | | ns | >0.9999 | |
| ZIP8^WT^ + PBS - *ZIP8^A391T^* + TNFα | -60 | -543.8 to 423.8 | | | | ns | >0.9999 | |
| ZIP8^WT^ + TNFα - *ZIP8^A391T^* + PBS | 40 | -443.8 to 523.8 | | | | ns | >0.9999 | |
| ZIP8^WT^ + TNFα - *ZIP8^A391T^* + TNFα | -40 | -523.8 to 443.8 | | | | ns | >0.9999 | |
| ZIP8^A391T^ + PBS - *ZIP8^A391T^* + TNFα | -80 | -563.8 to 403.8 | | | | ns | >0.9999 | |
|  |  |  | | | |  |  | |
| **TNFα (10 ng/μL)** | | | | | | | | |
| **Contrast** | **Mean Diff.** | **95.00% CI of diff.** | | | | **Summary** | **Adjusted P Value** | |
| WT+PBS - WT+TNFα | -1370 | -1854 to -886.2 | | | | **** | <0.0001 | |
| WT+PBS - KO+PBS | -50 | -533.8 to 433.8 | | | | ns | >0.9999 | |
| WT+PBS - KO+TNFα | -2310 | -2794 to -1826 | | | | **** | <0.0001 | |
| WT+PBS - *ZIP8^WT^* + PBS | -20 | -503.8 to 463.8 | | | | ns | >0.9999 | |
| WT+PBS - *ZIP8^WT^* + TNFα | -830 | -1314 to -346.2 | | | | **** | <0.0001 | |
| WT+PBS - *ZIP8^A391T^* + PBS | -10 | -493.8 to 473.8 | | | | ns | >0.9999 | |
| WT+PBS - *ZIP8^A391T^* + TNFα | -1450 | -1934 to -966.2 | | | | **** | <0.0001 | |
| WT+TNFα - KO+PBS | 1320 | 836.2 to 1804 | | | | **** | <0.0001 | |
| WT+TNFα - KO+TNFα | -940 | -1424 to -456.2 | | | | **** | <0.0001 | |
| WT+TNFα - *ZIP8^WT^* + PBS | 1350 | 866.2 to 1834 | | | | **** | <0.0001 | |
| WT+TNFα - *ZIP8^WT^* + TNFα | 540 | 56.21 to 1024 | | | | * | 0.0144 | |
| WT+TNFα - *ZIP8^A391T^* + PBS | 1360 | 876.2 to 1844 | | | | **** | <0.0001 | |
| WT+TNFα - *ZIP8^A391T^* + TNFα | -80 | -563.8 to 403.8 | | | | ns | >0.9999 | |
| KO+PBS - KO+TNFα | -2260 | -2744 to -1776 | | | | **** | <0.0001 | |
| KO+PBS - *ZIP8^WT^* + PBS | 30 | -453.8 to 513.8 | | | | ns | >0.9999 | |
| KO+PBS - *ZIP8^WT^* + TNFα | -780 | -1264 to -296.2 | | | | **** | <0.0001 | |
| KO+PBS - *ZIP8^A391T^* + PBS | 40 | -443.8 to 523.8 | | | | ns | >0.9999 | |
| KO+PBS - *ZIP8^A391T^* + TNFα | -1400 | -1884 to -916.2 | | | | **** | <0.0001 | |
| KO+TNFα - *ZIP8^WT^* + PBS | 2290 | 1806 to 2774 | | | | **** | <0.0001 | |
| KO+TNFα - *ZIP8^WT^* + TNFα | 1480 | 996.2 to 1964 | | | | **** | <0.0001 | |
| KO+TNFα - *ZIP8^A391T^* + PBS | 2300 | 1816 to 2784 | | | | **** | <0.0001 | |
| KO+TNFα - *ZIP8^A391T^* + TNFα | 860 | 376.2 to 1344 | | | | **** | <0.0001 | |
| ZIP8^WT^ + PBS - *ZIP8^WT^* + TNFα | -810 | -1294 to -326.2 | | | | **** | <0.0001 | |
| ZIP8^WT^ + PBS - *ZIP8^A391T^* + PBS | 10 | -473.8 to 493.8 | | | | ns | >0.9999 | |
| ZIP8^WT^ + PBS - *ZIP8^A391T^* + TNFα | -1430 | -1914 to -946.2 | | | | **** | <0.0001 | |
| ZIP8^WT^ + TNFα - *ZIP8^A391T^* + PBS | 820 | 336.2 to 1304 | | | | **** | <0.0001 | |
| ZIP8^WT^ + TNFα - *ZIP8^A391T^* + TNFα | -620 | -1104 to -136.2 | | | | ** | 0.0021 | |
| ZIP8^A391T^ + PBS - *ZIP8^A391T^* + TNFα | -1440 | -1924 to -956.2 | | | | **** | <0.0001 | |
|  |  |  | | | |  |  | |
| **TNFα (30 ng/μL)** | | | | | | | | |
| **Contrast** | **Mean Diff.** | **95.00% CI of diff.** | | | | **Summary** | **Adjusted P Value** | |
| WT+PBS - WT+TNFα | -3160 | -3644 to -2676 | | | | **** | <0.0001 | |
| WT+PBS - KO+PBS | 0 | -483.8 to 483.8 | | | | ns | >0.9999 | |
| WT+PBS - KO+TNFα | -5600 | -6084 to -5116 | | | | **** | <0.0001 | |
| WT+PBS - *ZIP8^WT^* + PBS | 20 | -463.8 to 503.8 | | | | ns | >0.9999 | |
| WT+PBS - *ZIP8^WT^* + TNFα | -2060 | -2544 to -1576 | | | | **** | <0.0001 | |
| WT+PBS - *ZIP8^A391T^* + PBS | 20 | -463.8 to 503.8 | | | | ns | >0.9999 | |
| WT+PBS - *ZIP8^A391T^* + TNFα | -3280 | -3764 to -2796 | | | | **** | <0.0001 | |
| WT+TNFα - KO+PBS | 3160 | 2676 to 3644 | | | | **** | <0.0001 | |
| WT+TNFα - KO+TNFα | -2440 | -2924 to -1956 | | | | **** | <0.0001 | |
| WT+TNFα - *ZIP8^WT^* + PBS | 3180 | 2696 to 3664 | | | | **** | <0.0001 | |
| WT+TNFα - *ZIP8^WT^* + TNFα | 1100 | 616.2 to 1584 | | | | **** | <0.0001 | |
| WT+TNFα - *ZIP8^A391T^* + PBS | 3180 | 2696 to 3664 | | | | **** | <0.0001 | |
| WT+TNFα - *ZIP8^A391T^* + TNFα | -120 | -603.8 to 363.8 | | | | ns | >0.9999 | |
| KO+PBS - KO+TNFα | -5600 | -6084 to -5116 | | | | **** | <0.0001 | |
| KO+PBS - *ZIP8^WT^* + PBS | 20 | -463.8 to 503.8 | | | | ns | >0.9999 | |
| KO+PBS - *ZIP8^WT^* + TNFα | -2060 | -2544 to -1576 | | | | **** | <0.0001 | |
| KO+PBS - *ZIP8^A391T^* + PBS | 20 | -463.8 to 503.8 | | | | ns | >0.9999 | |
| KO+PBS - *ZIP8^A391T^* + TNFα | -3280 | -3764 to -2796 | | | | **** | <0.0001 | |
| KO+TNFα - *ZIP8^WT^* + PBS | 5620 | 5136 to 6104 | | | | **** | <0.0001 | |
| KO+TNFα - *ZIP8^WT^* + TNFα | 3540 | 3056 to 4024 | | | | **** | <0.0001 | |
| KO+TNFα - *ZIP8^A391T^* + PBS | 5620 | 5136 to 6104 | | | | **** | <0.0001 | |
| KO+TNFα - *ZIP8^A391T^* + TNFα | 2320 | 1836 to 2804 | | | | **** | <0.0001 | |
| ZIP8^WT^ + PBS - *ZIP8^WT^* + TNFα | -2080 | -2564 to -1596 | | | | **** | <0.0001 | |
| ZIP8^WT^ + PBS - *ZIP8^A391T^* + PBS | 0 | -483.8 to 483.8 | | | | ns | >0.9999 | |
| ZIP8^WT^ + PBS - *ZIP8^A391T^* + TNFα | -3300 | -3784 to -2816 | | | | **** | <0.0001 | |
| ZIP8^WT^ + TNFα - *ZIP8^A391T^* + PBS | 2080 | 1596 to 2564 | | | | **** | <0.0001 | |
| ZIP8^WT^ + TNFα - *ZIP8^A391T^* + TNFα | -1220 | -1704 to -736.2 | | | | **** | <0.0001 | |
| ZIP8^A391T^ + PBS - *ZIP8^A391T^* + TNFα | -3300 | -3784 to -2816 | | | | **** | <0.0001 | |
|  |  |  | | | |  |  | |
| **TNFα (100 ng/μL)** | | | | | | | | |
| **Contrast** | **Mean Diff.** | **95.00% CI of diff.** | | | | **Summary** | **Adjusted P Value** | |
| WT+PBS - WT+TNFα | -3360 | -3844 to -2876 | | | | **** | <0.0001 | |
| WT+PBS - KO+PBS | -30 | -513.8 to 453.8 | | | | ns | >0.9999 | |
| WT+PBS - KO+TNFα | -5680 | -6164 to -5196 | | | | **** | <0.0001 | |
| WT+PBS - *ZIP8^WT^* + PBS | 30 | -453.8 to 513.8 | | | | ns | >0.9999 | |
| WT+PBS - *ZIP8^WT^* + TNFα | -2330 | -2814 to -1846 | | | | **** | <0.0001 | |
| WT+PBS - *ZIP8^A391T^* + PBS | 0 | -483.8 to 483.8 | | | | ns | >0.9999 | |
| WT+PBS - *ZIP8^A391T^* + TNFα | -3240 | -3724 to -2756 | | | | **** | <0.0001 | |
| WT+TNFα - KO+PBS | 3330 | 2846 to 3814 | | | | **** | <0.0001 | |
| WT+TNFα - KO+TNFα | -2320 | -2804 to -1836 | | | | **** | <0.0001 | |
| WT+TNFα - *ZIP8^WT^* + PBS | 3390 | 2906 to 3874 | | | | **** | <0.0001 | |
| WT+TNFα - *ZIP8^WT^* + TNFα | 1030 | 546.2 to 1514 | | | | **** | <0.0001 | |
| WT+TNFα - *ZIP8^A391T^* + PBS | 3360 | 2876 to 3844 | | | | **** | <0.0001 | |
| WT+TNFα - *ZIP8^A391T^* + TNFα | 120 | -363.8 to 603.8 | | | | ns | >0.9999 | |
| KO+PBS - KO+TNFα | -5650 | -6134 to -5166 | | | | **** | <0.0001 | |
| KO+PBS - *ZIP8^WT^* + PBS | 60 | -423.8 to 543.8 | | | | ns | >0.9999 | |
| KO+PBS - *ZIP8^WT^* + TNFα | -2300 | -2784 to -1816 | | | | **** | <0.0001 | |
| KO+PBS - *ZIP8^A391T^* + PBS | 30 | -453.8 to 513.8 | | | | ns | >0.9999 | |
| KO+PBS - *ZIP8^A391T^* + TNFα | -3210 | -3694 to -2726 | | | | **** | <0.0001 | |
| KO+TNFα - *ZIP8^WT^* + PBS | 5710 | 5226 to 6194 | | | | **** | <0.0001 | |
| KO+TNFα - *ZIP8^WT^* + TNFα | 3350 | 2866 to 3834 | | | | **** | <0.0001 | |
| KO+TNFα - *ZIP8^A391T^* + PBS | 5680 | 5196 to 6164 | | | | **** | <0.0001 | |
| KO+TNFα - *ZIP8^A391T^* + TNFα | 2440 | 1956 to 2924 | | | | **** | <0.0001 | |
| ZIP8^WT^ + PBS - *ZIP8^WT^* + TNFα | -2360 | -2844 to -1876 | | | | **** | <0.0001 | |
| ZIP8^WT^ + PBS - *ZIP8^A391T^* + PBS | -30 | -513.8 to 453.8 | | | | ns | >0.9999 | |
| ZIP8^WT^ + PBS - *ZIP8^A391T^* + TNFα | -3270 | -3754 to -2786 | | | | **** | <0.0001 | |
| ZIP8^WT^ + TNFα - *ZIP8^A391T^* + PBS | 2330 | 1846 to 2814 | | | | **** | <0.0001 | |
| ZIP8^WT^ + TNFα - *ZIP8^A391T^* + TNFα | -910 | -1394 to -426.2 | | | | **** | <0.0001 | |
| ZIP8^A391T^ + PBS - *ZIP8^A391T^* + TNFα | -3240 | -3724 to -2756 | | | | **** | <0.0001 | |
|  |  |  | | | |  |  | |
| **TNFα (300 ng/μL)** | | | | | | | | |
| **Contrast** | **Mean Diff.** | **95.00% CI of diff.** | | | | **Summary** | **Adjusted P Value** | |
| WT+PBS - WT+TNFα | -3580 | -4064 to -3096 | | | | **** | <0.0001 | |
| WT+PBS - KO+PBS | -20 | -503.8 to 463.8 | | | | ns | >0.9999 | |
| WT+PBS - KO+TNFα | -6590 | -7074 to -6106 | | | | **** | <0.0001 | |
| WT+PBS - *ZIP8^WT^* + PBS | 30 | -453.8 to 513.8 | | | | ns | >0.9999 | |
| WT+PBS - *ZIP8^WT^* + TNFα | -2820 | -3304 to -2336 | | | | **** | <0.0001 | |
| WT+PBS - *ZIP8^A391T^* + PBS | 0 | -483.8 to 483.8 | | | | ns | >0.9999 | |
| WT+PBS - *ZIP8^A391T^* + TNFα | -3870 | -4354 to -3386 | | | | **** | <0.0001 | |
| WT+TNFα - KO+PBS | 3560 | 3076 to 4044 | | | | **** | <0.0001 | |
| WT+TNFα - KO+TNFα | -3010 | -3494 to -2526 | | | | **** | <0.0001 | |
| WT+TNFα - *ZIP8^WT^* + PBS | 3610 | 3126 to 4094 | | | | **** | <0.0001 | |
| WT+TNFα - *ZIP8^WT^* + TNFα | 760 | 276.2 to 1244 | | | | **** | <0.0001 | |
| WT+TNFα - *ZIP8^A391T^* + PBS | 3580 | 3096 to 4064 | | | | **** | <0.0001 | |
| WT+TNFα - *ZIP8^A391T^* + TNFα | -290 | -773.8 to 193.8 | | | | ns | >0.9999 | |
| KO+PBS - KO+TNFα | -6570 | -7054 to -6086 | | | | **** | <0.0001 | |
| KO+PBS - *ZIP8^WT^* + PBS | 50 | -433.8 to 533.8 | | | | ns | >0.9999 | |
| KO+PBS - *ZIP8^WT^* + TNFα | -2800 | -3284 to -2316 | | | | **** | <0.0001 | |
| KO+PBS - *ZIP8^A391T^* + PBS | 20 | -463.8 to 503.8 | | | | ns | >0.9999 | |
| KO+PBS - *ZIP8^A391T^* + TNFα | -3850 | -4334 to -3366 | | | | **** | <0.0001 | |
| KO+TNFα - *ZIP8^WT^* + PBS | 6620 | 6136 to 7104 | | | | **** | <0.0001 | |
| KO+TNFα - *ZIP8^WT^* + TNFα | 3770 | 3286 to 4254 | | | | **** | <0.0001 | |
| KO+TNFα - *ZIP8^A391T^* + PBS | 6590 | 6106 to 7074 | | | | **** | <0.0001 | |
| KO+TNFα - *ZIP8^A391T^* + TNFα | 2720 | 2236 to 3204 | | | | **** | <0.0001 | |
| ZIP8^WT^ + PBS - *ZIP8^WT^* + TNFα | -2850 | -3334 to -2366 | | | | **** | <0.0001 | |
| ZIP8^WT^ + PBS - *ZIP8^A391T^* + PBS | -30 | -513.8 to 453.8 | | | | ns | >0.9999 | |
| ZIP8^WT^ + PBS - *ZIP8^A391T^* + TNFα | -3900 | -4384 to -3416 | | | | **** | <0.0001 | |
| ZIP8^WT^ + TNFα - *ZIP8^A391T^* + PBS | 2820 | 2336 to 3304 | | | | **** | <0.0001 | |
| ZIP8^WT^ + TNFα - *ZIP8^A391T^* + TNFα | -1050 | -1534 to -566.2 | | | | **** | <0.0001 | |
| ZIP8^A391T^ + PBS - *ZIP8^A391T^* + TNFα | -3870 | -4354 to -3386 | | | | **** | <0.0001 | |
